# A Multidimensional Electronic Hydroxyurea Adherence Intervention for Children With Sickle Cell Disease: Single-Arm Before-After Study

**DOI:** 10.2196/13452

**Published:** 2019-08-08

**Authors:** Susan Creary, Deena Chisolm, Joseph Stanek, Jane Hankins, Sarah H O'Brien

**Affiliations:** 1 Nationwide Children's Hospital The Ohio State University Columbus, OH United States; 2 St Jude Children's Research Hospital Memphis, TN United States

**Keywords:** hydroxyurea, children, sickle cell disease, mobile health, mhealth, adherence, textmessaging

## Abstract

**Background:**

Hydroxyurea is a disease-modifying medication for patients with sickle cell disease (SCD). Despite demonstrated efficacy, hydroxyurea nonadherence in clinical practice is common and results in worse health outcomes for nonadherent patients. Mobile Directly Observed Therapy (Mobile DOT) is a pilot-tested, electronic, multidimensional hydroxyurea adherence intervention for children with SCD. Mobile DOT includes sending daily text message reminders to patients to take hydroxyurea, patients recording and sending daily videos that capture their hydroxyurea administrations for the research team to review and track adherence, providing personalized feedback to patients about their adherence, and providing small monetary incentives to patients if they achieve high hydroxyurea adherence.

**Objective:**

This study aimed to determine if Mobile DOT increases hydroxyurea adherence in children with SCD and to explore its impact on hematologic and clinical outcomes.

**Methods:**

This was a single-arm, 6-month intervention study of patients with SCD on hydroxyurea who were aged ≤19 years and reported having access to an electronic device. Participants’ hydroxyurea adherence when they received Mobile DOT was compared with their adherence 6 months before and after receiving Mobile DOT. Participants’ medication possession ratio (MPR) was calculated from their pharmacy dispensing records and was used to measure adherence. Laboratory and clinical outcomes were abstracted from participants’ electronic medical records. Infrequently hospitalized patients who received at least 160 days of the intervention were considered to be engaged participants.

**Results:**

Of 91 patients who were approached, 55 enrolled and 34 engaged with Mobile DOT. The median age of the engaged participants was 10 years (range 2-18.8 years), and 21 (62%, 21/34) participants were male, 28 (82%, 21/34) had hemoglobin SS SCD, and 19 (56%, 19/34) were prescribed hydroxyurea for at least a year before enrollment. With Mobile DOT, engaged participants’ median MPR increased from 61.7% to 84.4% (*P*<.001) and significantly more (67% vs 30%; *P*=.002) achieved ≥80% hydroxyurea adherence compared with baseline values. Engaged participants’ mean fetal hemoglobin (HgbF) levels and mean corpuscular volumes (MCV) improved significantly after 6 months of Mobile DOT (*P*=.04 and *P*=.001, respectively), but their adherence, HgbF levels, and MCV returned to baseline values during the 6 months after the intervention. Hospitalizations and the clinical outcomes that were measured occurred infrequently during the study. Nonengagement was associated with being female and having a recent SCD complication. In addition, having insufficient electronic data, being unable to quickly complete Mobile DOT each day, and not perceiving that Mobile DOT was beneficial may have further decreased engagement.

**Conclusions:**

Mobile DOT shows promise as an effective intervention for some children with SCD. Modifications that may improve recruitment, reduce attrition, and increase engagement were identified and could increase the impact that Mobile DOT has on children with SCD.

**Trial Registration:**

ClinicalTrials.gov NCT02578017; https://clinicaltrials.gov/ct2/show/NCT02578017

## Introduction

Sickle cell disease (SCD) is the most common, severe inherited blood disorder. Children with SCD experience substantial morbidity, including frequent vaso-occlusive crisis (VOC) pain and acute chest syndrome (ACS) episodes, stroke, and decreased quality of life [1,2]. Hydroxyurea is a once-daily, Food and Drug Administration–approved, preventative, and disease-modifying medication for children with SCD. Multiple large studies show that hydroxyurea improves quality of life while reducing the frequency of VOC and ACS episodes, hospitalizations, hospital readmissions, and transfusions in children with SCD [3-5]. Despite demonstrated efficacy and safety, hydroxyurea nonadherence is common in clinical practice. Health insurance claims data suggest that only 30% to 50% of children with SCD on hydroxyurea in the United States take it on at least 80% of the days it is prescribed, and this nonadherence is associated with worse clinical outcomes, lower quality of life, and higher costs of care for nonadherent patients [2,6,7].

Strategies to improve adherence to medications for other chronic diseases include interventions such as directly observed therapy for tuberculosis [8], reminder alerts for hyperlipidemia and hypertension [9], counseling or motivational interviewing for human immunodeficiency virus [10], and incentives or contingency management for diabetes [11]. In children and adolescents with chronic diseases, including those with SCD, electronic devices show promise as useful tools to provide these interventions to improve adherence and disease self-management [12-15]. Single-dimension adherence interventions, however, have inherent limitations. They also show varying levels of success at increasing adherence likely because they are not able to address multiple components of the Health Behavior Model (HBM), the health promotion theory frequently used to explain medication adherence behavior [16]. The HBM proposes that perceived disease severity, susceptibility to disease complications, self-efficacy, costs and benefits of being adherent, and receiving reminder cues are factors that influence adherence. For example, in the context of the HBM, hydroxyurea nonadherence could occur if patients and/or their families do not perceive the long-term health benefits of taking hydroxyurea outweigh the potential risk of experiencing side effects or the length of time that they must take hydroxyurea before its benefits begin to manifest. In addition, for young children with SCD who rely on caregivers to be adherent, nonadherence could result if caregivers do not have the self-efficacy skills to (1) remember to administer the hydroxyurea when it is due, (2) accurately measure the correct dose of hydroxyurea, and/or (3) deliver hydroxyurea to their children if they are uncooperative with administration. Finally, for adolescents with SCD, perceived invulnerability or insusceptibility, which are common during this stage of development, could contribute to hydroxyurea nonadherence and negative health outcomes.

Therefore, to address multiple HBM constructs that can influence hydroxyurea adherence behavior using devices that most children with SCD report having access to [17], we pilot tested an electronic, multidimensional adherence strategy called Mobile Directly Observed Therapy (Mobile DOT) and published these results in 2014 [18]. Mobile DOT is a hydroxyurea adherence strategy that includes sending daily text message (short message service [SMS]) to patients to remind them to take hydroxyurea, patients recording and sending daily videos that capture their hydroxyurea administrations to the research team to review and track adherence, electronically providing personalized feedback to patients about their adherence, and providing small monetary incentives to patients if they achieve high hydroxyurea adherence. In our pilot work, we found that Mobile DOT was a feasible and acceptable adherence intervention and could achieve ≥90% hydroxyurea adherence in a small cohort of children with SCD. The objectives of this study were to determine if Mobile DOT increases hydroxyurea adherence and to explore its potential impact on hematologic and clinical outcomes in a larger population of children with SCD.

## Methods

### Study Design, Recruitment, and Participants

These data were collected as part of an institutional review board–approved study. The details of the Mobile DOT intervention and the protocol for this study were previously published [19]. Briefly, this was a single-arm, cross-over study performed at Nationwide Children’s Hospital (NCH), a large, comprehensive pediatric hospital in Ohio. English-speaking patients with SCD (any genotype) who were aged ≤19 years, prescribed hydroxyurea for at least the previous 180 days, not receiving chronic transfusion therapy, planned to receive care at NCH for the following year, and had (or their legal guardian had) personal daily access to an electronic device (eg, smartphone or tablet) that could receive SMS text message alerts and record and send daily videos were eligible. Electronic device access was confirmed, before enrollment, when the patient successfully submitted a video to the secure Mobile DOT website.

Patients were sequentially approached to participate when they presented for clinical care, and follow-up study visits were completed during participants’ standard of care hematology visits. Participants’ electronic medical records (EMRs) were used to collect demographic information, laboratory and clinical data, hydroxyurea dispensing pharmacies, and duration of hydroxyurea treatment. Hydroxyurea dosing and monitoring were left to clinicians’ discretion and tracked using the EMR, but institutional standards, derived from published hydroxyurea protocols [20], were available to guide this management.

There were 3 sequential study periods: baseline, Mobile DOT, and postintervention that were each approximately 180 days. The baseline period was defined as the 180 days before enrollment, the Mobile DOT period started the day following enrollment, and the postintervention period started immediately after the Mobile DOT period. The Mobile DOT and postintervention periods were designed to be 180 days but could vary slightly in length to coordinate participants’ study visits with their standard of care hematology visits. In addition, because hydroxyurea administration that occurs in the hospital setting is highly monitored and includes interventions that are separate from Mobile DOT (eg, all hydroxyurea dose administrations are observed and tracked by a nurse), only participants who completed at least 160 days of the Mobile DOT period and who were hospitalized for fewer than 20 days during this time were considered to be engaged participants. Participants who completed at least 160 days of the postintervention period before their final study visit and were hospitalized for fewer than 20 days during this period were considered to have completed the study.

### Mobile Directly Observed Therapy

A custom and secure Mobile DOT website to send the alerts and store participants’ videos was built by the research technology team at NCH and run under an Apache Web server (version 2.2.15; Apache Software Foundation), was written in the Perl and JavaScript languages, and was run on a 64-bit CentOS 6.5 Linux operating system. Participants were informed before consenting of the potential risk that videos could potentially be intercepted during transmission to the secure website, but their videos were secure once they were received by the website.

#### Alerts

Participants identified their preferred hydroxyurea administration time and developed their own personalized SMS text message alerts at enrollment. Using a secure Microsoft Exchange mail server, participants and/or their consenting caregivers (if aged <14 years) were sent up to 4 SMS text message alerts to remind them to take hydroxyurea every day.

#### Video Directly Observed Therapy

Participants were instructed to self-record (or have their consenting caregiver record if aged<11 years) their daily hydroxyurea administrations with their electronic device and email these videos as an attachment every day to the secure website for the research team to review. Participants were trained to record continuous videos that allowed for easy participant recognition, a view of their hydroxyurea before and during administration, and a view of their empty mouth after ingestion. Participants who were instructed to temporarily discontinue hydroxyurea by their clinical provider (eg, for myelosuppression) were instructed to continue to submit daily videos, stating this information to be considered adherent during this time. VDOT was not required during hospitalizations. Temporary electronic device access lapses (for up to 5 days for each 60-day segment of the intervention period) were allowed, but to be considered adherent during these lapses, participants had to notify the research team (by email, SMS text message, or voicemail) on each of these days to confirm that they had taken their hydroxyurea.

A background script on the secure server retrieved the participants’ emails with video attachments. Videos were stored locally on the operating system in a participant directory with restricted access to the research team. Information about the participants, the emails, and videos were stored in a local SQLite database. To ensure that the videos were unique, the research team reviewed all videos to ensure that they represented valid hydroxyurea administration and checked the megabyte size of the videos to ensure variability if there was concern that participants were submitting videos that were not unique. Only 2 research team members who were familiar with the participants were responsible for reviewing and tracking adherence.

#### Feedback

The research team attempted to contact participants (or their consenting caregiver) through SMS text message, email, or telephone when a video was not received to encourage daily hydroxyurea adherence and provide positive feedback when participants achieved the adherence incentive goal.

#### Incentives

Participants were mailed a US $30 gift card if their videos confirmed at least 90% adherence over the past 30 days. A research team member spent approximately 1 min per participant per day to track adherence and send messages when needed.

### Outcome Measures

#### Medication Possession Ratio

MPR is the percentage of days during a given period of time that patients have access to a medication. MPR was the primary adherence measure used because the pharmacy dispensing data needed for this calculation could be retrospectively collected. Previous studies suggest if multiple months of pharmacy dispensing records are used, MPR provides an accurate quantitative estimate of patients’ maximum possible adherence during that time [21-23]. Hydroxyurea dispensing records were requested after the participant completed their participation in the study and from all pharmacies that participants’ self-reported they used and/or were identified in their EMR. Participants’ hydroxyurea MPR was calculated for each study period using the following formula: the number of days that dispensing records confirmed access to hydroxyurea in a study period divided by the total number of days in a study period, multiplied by 100. MPR calculations accounted for hydroxyurea doses that were held or received during any hospitalization and for any prescribed dose adjustments documented in the EMR during the study. Participants’ with a hydroxyurea MPR of ≥80% during a study period were defined as adherent for that period because improved clinical outcomes were seen at this adherence threshold in a randomized pediatric SCD hydroxyurea clinical trial [3,24].

#### Video Directly Observed Therapy Adherence

Observed adherence, either in-person or by video, is a reliable and valid measure of patients’ medication adherence [21]. Engaged participants’ VDOT adherence was calculated using the following formula: the number of days VDOT was completed divided by the total number of days in the Mobile DOT period multiplied by 100. VDOT adherence calculations accounted for days that the research team was notified of device access lapse and for doses received during hospitalizations.

#### Laboratory Data

Large pediatric clinical trials show that hydroxyurea exposure significantly increases fetal hemoglobin (HgbF) production, total hemoglobin (Hgb), and mean corpuscular volume (MCV) and reduces absolute neutrophil counts (ANC) [3,25-27]. In this study, laboratory studies that were obtained during standard of care hematology visits within 40 days of the end of a study period were analyzed. NCH uses Sebia Zone Electrophoresis to reliably quantify HgbF levels [28] and the Sysmex XN-1000, a standard hematology analyzer, to reliably measure Hgb, MCV, and ANC [29].

#### Clinical Outcomes

Multiple pediatric clinical trials confirm that hydroxyurea reduces hospitalizations, erythrocyte transfusions, ACS episodes, and VOC episodes requiring hospitalization [3,30]. These outcomes were abstracted from participants’ EMR during the study.

#### Satisfaction Survey

To measure the amount of time that it took participants to complete Mobile DOT each day, their willingness to continue the intervention without payment, and their satisfaction and perceived effectiveness of the intervention, participants (or the consenting caregivers of participants aged<14 years) completed the 5-point Likert scale Mobile DOT satisfaction survey [18] at the end of their Mobile DOT period or at their withdrawal visit. Participants who withdrew were also asked to provide a reason for withdrawal.

### Study Withdrawal

Participants who discontinued hydroxyurea moved out of the area or informed the research team that they did not want to continue to participate were immediately withdrawn from the study. The research team also stopped sending SMS text message alerts and attempting to contact participants who did not submit a video or respond to research staff communications for more than 30 consecutive days during the Mobile DOT period. These participants were withdrawn from the study at their next clinic visit.

### Statistical Analysis

Descriptive statistics were used to describe sample characteristics and report the clinical outcomes, frequency and percentages for qualitative variables, and median and range or mean and standard error for quantitative variables. Nonparametric tests were used to compare baseline characteristics between engaged and nonengaged participants. McNemar test was used to compare proportion of patients who were adherent at baseline and with Mobile DOT. To compare adherence and hematologic measures across the 3 study periods, linear mixed models with Tukey-adjusted pairwise comparisons were used. All *P* values were 2-sided, and *P*<.05 was considered statistically significant. Statistical analyses were performed using SAS software, version 9.4 (SAS Institute).

## Results

### Study Participants

**Figure 1:**
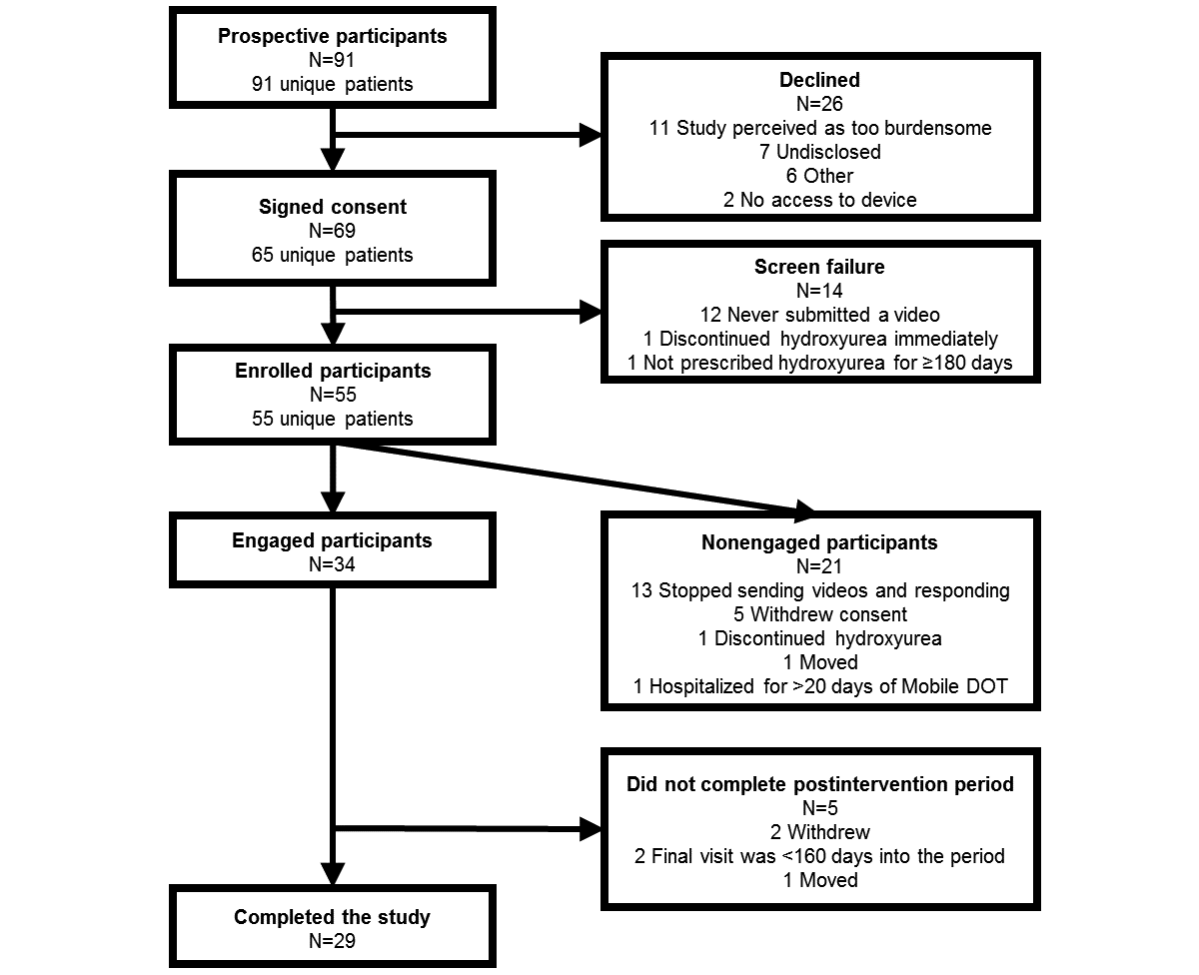
Participant flow diagram (Mobile DOT: Mobile Directly Observed Therapy).

Between July 2014 and January 2017, 91 unique prospective participants were approached, 55 enrolled (76% of the planned sample size), 34 engaged with the Mobile DOT intervention, and 29 completed the study (Figure 1). All the participants planned to use a smartphone as their electronic device. None of the patients who declined participation reported privacy concerns as a reason for study refusal. Recruitment ended before enrolling the planned sample size when 97% of the prospective patients had been approached because recruitment would have needed to be extended for multiple months to approach the remaining patients at their standard hematology visits.

Dispensing records were received for 54 (33 engaged) participants, and these participants were included in the MPR analyses. At baseline, 14 (25%, 14/54) enrolled participants were adherent (MPR ≥80%) to hydroxyurea, and 17 (31%, 17/54) had a hydroxyurea MPR <50%.

Table 1 shows a comparison of the baseline characteristics between engaged and nonengaged participants. Of the 17 engaged participants who were aged<10 years, 8 (47%) were adherent to hydroxyurea at baseline and 3 (27%) of the 11 nonengaged participants who were aged<10 years were adherent at baseline. In addition, 2 (13%) of the 17 engaged participants who were aged ≥10 years were adherent to hydroxyurea at baseline, and one (10%) of the 10 nonengaged participants who were aged ≥10 years was adherent to hydroxyurea at baseline. Engaged participants received Mobile DOT for a median of 191 days, and those who completed the study were followed for a median of 181 days during the postintervention period. Engaged participants’ median prescribed hydroxyurea dose did not significantly change from baseline to the end of the Mobile DOT period (25.9 mg/kg/day vs 25.8 mg/kg/day; *P*>.99). Of those who completed the study, 21 (72%) were prescribed a dose that was within 5 mg/kg/day of their baseline dose, but their median prescribed hydroxyurea dose was significantly lower at the end of the postintervention period compared with baseline (23.5 mg/kg/day; *P*=.004).

**Table 1:** Participants’ baseline characteristics.

Characteristics		All (n=55)	Nonengaged (n=21)	Engaged (n=34)	*P* value
Median age (years; range)		10.0 (2.0-19.0)	9.4 (2.7-19.0)	10.0 (2.0-18.8)	.95
**Age (years), n (%)**					.99
	<10	28 (51)	11 (52)	17 (50)	
	Oct-17	22 (40)	8 (38)	14 (41)	
	≥18	5	2	3	
Male, n (%)		26 (47)	5 (234)	21 (62)	.01
**Race, n (%)**					.52
	Black or African American	53 (96)	21 (100)	32 (94)	
	Biracial	2	0	2	
**Genotype, n (%)**					.93
	SS	47 (856)	19 (91)	28 (82)	
	SC	3 (6)	1 (5)	2 (6)	
	SB^0^	2 (4)	1 (5)	1 (3)	
	SB^+^	2 (4)	0 (0)	2 (6)	
	Other (SE)	1 (2)	0 (0)	1 (3)	
**Duration of hydroxyurea use, n (%)**					.8
	6-12 months	25 (456)	10 (48)	15 (44)	
	>12 months	30 (55)	11 (52)	19 (56)	
**Indication for hydroxyurea, n (%)**					
	Clinical complication	53 (96)	21 (100)	32 (94)	.52
	Severe genotype or family request^a^	4 (7)	1 (5)	3 (9)	.99
Median hydroxyurea dose (mg/kg/d)		25.9	25.9	25.9	.41
**Hydroxyurea MPR^b^**					
	Median, %	61.7	66.7	61.1	.86
	0%-19%, n	3	1	2	—^c^
	20%-39%, n	9	6	3	—
	40%-59%, n	11	1	10	—
	60%-79%, n	17	9	8	—
	≥80%, n	14	4	10	—
**Median hematologic studies**					
	Hemoglobin, g/dL	9.3	8.6	9.4	.14
	MCV^d^, fl/L	90.9	91.8	88.2	.68
	Hemoglobin F, %	21.1	17.8	24.8	.07
	Absolute neutrophil count per µL	3690	4770	3415	.003

^a^Initiated hydroxyurea therapy before experiencing a sickle cell disease complication.

^b^Medication possession ratio (MPR) could not be calculated for 1 participant because dispensing records were not received.

^c^Descriptive data only.

^d^MCV: mean corpuscular volume.

### Hydroxyurea Adherence

**Figure 2:**
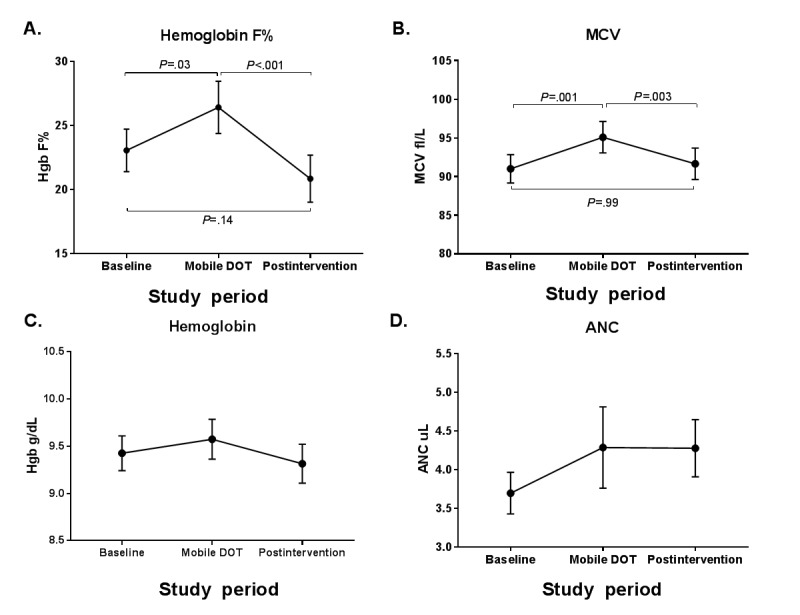
Participants’ hydroxyurea MPR adherence during each study period (Mobile DOT: Mobile Directly Observed Therapy; MPR: medication possession ratio).

Participants’ median hydroxyurea MPR was significantly higher with Mobile DOT compared with baseline, but median postintervention MPR was not significantly different compared with baseline (Figure 2). The proportion of engaged participants that were adherent (MPR ≥80%) to hydroxyurea significantly increased from 30% at baseline to 67% with Mobile DOT (*P*=.002). In addition, 20 (61%) of the engaged participants increased their MPR by >15% with Mobile DOT compared with their baseline. Median VDOT adherence (88.1%) and MPR adherence (87.8%) for the engaged participants during the Mobile DOT period were not significantly different (*P*=.99).

### Hematologic and Clinical Outcomes

**Figure 3:**
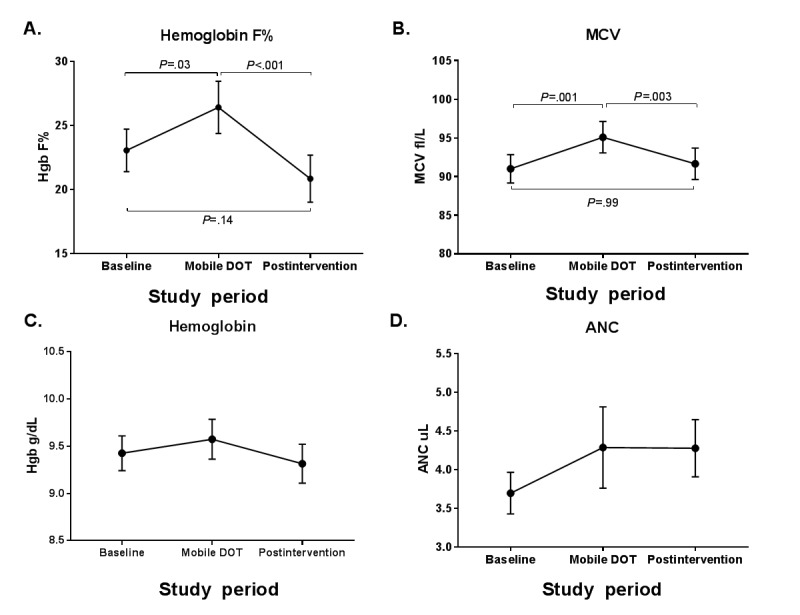
Engaged participants’ (n=34) laboratory studies at the end of each study period (ANC: absolute neutrophil count; Hgb: hemoglobin; MCV: mean corpuscular volume; Mobile DOT: Mobile Directly Observed Therapy).

Engaged participants’ mean MCV and HgbF levels at the end of Mobile DOT were significantly higher than baseline but were not significantly different at the end of postintervention period compared with baseline. Engaged participants’ mean Hgb and ANC did not significantly change throughout the study (Figure 3).

During the baseline period, 8 participants received at least one erythrocyte transfusion (6 nonengaged vs 2 engaged, *P*=.04), 11 had at least one ACS episode (8 nonengaged vs 3 engaged, *P*=.01), and 12 had at least one VOC hospitalization (7 nonengaged vs 5 engaged, *P*=.18). The cohort experienced a total of 13 ACS episodes and 26 VOC hospitalizations during the baseline period, but 1 participant accounted for 9 of these VOC hospitalizations and was nonengaged during the Mobile DOT period because of continued recurrent VOC hospitalizations. During the Mobile DOT period, 3 engaged participants received at least one erythrocyte transfusion, 2 had at least one ACS episode, and 6 had at least one VOC hospitalization. During the postintervention period, 1 participant required at least one transfusion, 3 had at least one ACS episode, and 3 had at least one VOC hospitalization.

### Satisfaction Survey

All the engaged participants and 16 (76%) of the nonengaged participants completed the satisfaction survey. Of the engaged participants, 31 (92%) agreed that they took hydroxyurea more often with Mobile DOT, 34 (100%) agreed that Mobile DOT was easy to use, 30 (88%) agreed that they would be willing to continue Mobile DOT, 19 (56%) agreed that they would be willing to continue Mobile DOT without pay, and 30 (88%) reported they could complete Mobile DOT in less than 3 min each day. Of the nonengaged participants, 9 (56%) agreed that they took hydroxyurea more often with Mobile DOT, 8 (50%) agreed that Mobile DOT was easy to use, 7 (44%) agreed they would be willing to continue Mobile DOT, 5 (31%) agreed that they would be willing to continue Mobile DOT without pay, and 8 (50%) reported that they were able to complete Mobile DOT in less than 3 min each day.

## Discussion

### Hydroxyurea Adherence

Hydroxyurea nonadherence is common among children with SCD [7,24], and an effective hydroxyurea adherence intervention is needed to improve health outcomes. Overall, our results suggest that Mobile DOT improves hydroxyurea adherence, at least temporarily, in children who engage with the intervention. Of the engaged children, two-thirds achieved 80% or higher MPR adherence with Mobile DOT, which is noteworthy considering many of these children had appreciably lower baseline hydroxyurea adherence. Also, median MPR significantly improved with Mobile DOT, despite including some subjects with less potential to improve their MPR because they had 80% or higher adherence at baseline. Finally, although not all the engaged participants achieved 80% or higher adherence with Mobile DOT, many still incrementally increased their adherence (eg, >15% increase or an additional hydroxyurea dose per week), suggesting that Mobile DOT has the potential to improve adherence for some of the most nonadherent children.

### Hematologic Outcomes

Our results also showed that engaged participants’ mean MCV and HgbF levels increased during the Mobile DOT period. Given that MCV and HgbF level changes from hydroxyurea exposure typically occur before clinical improvements manifest [30], this could be an early indication that with continued use, Mobile DOT may have potential hematologic and clinical benefits. Although significant Hgb changes were not appreciated, this may be related to the relatively short duration of time that Mobile DOT was provided compared with the time required to see improved Hgb with hydroxyurea [3] and our participants’ relatively high baseline Hgb levels. In addition, 15% of our engaged patients had either Hemoglobin SC, Sβ^+^, or SE, and past studies suggest that other SCD genotypes may not have the same degree of hematologic response with hydroxyurea compared with those with hemoglobin SS [31,32].

Similar to other medication adherence interventions [33], participants’ adherence and laboratory improvements declined once the intervention was discontinued. Survey results showed that many engaged participants were willing to continue Mobile DOT beyond 6 months, suggesting that for some, Mobile DOT may be able to be used longer term to sustain adherence. However, short booster sessions or additional interventions may be needed for others when their adherence declines. Laboratory improvements likely returned to baseline because adherence declined but also the median prescribed hydroxyurea dose decreased by the end of the study. Mean ANC did not change during the study to suggest that severe myelosuppression frequently occurred that would have necessitated this decrease. Most participants were also prescribed doses at the end of the study that were similar to their baseline doses. We, therefore, suspect this decrease was because many participants were growing children and the hydroxyurea protocol [20] and institutional guidelines do not provide guidance about how frequently to increase hydroxyurea to account for weight gain from growth.

### Clinical Outcomes

Future studies are needed to definitively determine if Mobile DOT reduces erythrocyte transfusions, hospitalizations, and ACS and VOC episodes because these events occurred infrequently at baseline and participants were followed for a relatively short period of time. To limit underestimating the benefit of effective adherence interventions, future studies should also measure their impact on preventing other patient-reported and costly outcomes, such as VOC episodes, that do not result in hospitalization [34] and stroke [35] that may be ameliorated or prevented with hydroxyurea.

### Limitations

Several limitations must be considered when interpreting our results. First, despite approaching almost all the eligible patients at NCH, we were unable to enroll our projected sample size [19]. This occurred because we anticipated that more patients at NCH were going to initiate hydroxyurea and become eligible than actually did and because some patients perceived the intervention as too burdensome. These findings underscore the need for interventions, such as shared decision-making tools [36], to increase hydroxyurea acceptance and add to the existing literature that suggests that the time and burden to participate in clinical studies for patients with SCD are prohibitive [37]. In the future, we plan to recruit patients outside of the clinical care setting to avoid approaching patients when they may be overwhelmed by other aspects of their SCD care. This strategy could also increase the number of nonadherent patients that are recruited because clinic nonadherence is associated with hydroxyurea nonadherence [38].

Second, only two-thirds of the enrolled participants remained engaged with Mobile DOT. As only those who engaged improved their adherence, it is possible that the factors that promoted engagement may have contributed to the observed adherence gains. Therefore, identifying these mediating factors is paramount to increasing the impact that this and other adherence interventions may have on children with SCD. For example, our results suggest that nonengaged participants were more likely to be female and have experienced a recent SCD complication (eg, erythrocyte transfusion and/or ACS episode) compared with engaged participants. The small sample size and small number of clinical complications that occurred during the study limit the ability to definitively determine if these factors influence engagement, if they were related to an unmeasured moderating factor, or if they were merely a random finding observed in this sample. In addition, consistent with the literature that suggests widespread electronic device ownership among African Americans [39], including children with SCD [13], only 3 patients reported that not owning a device prevented them from participating or led to their study withdrawal. However, increasing literature suggests that having an insufficient data plan or inconsistent access to Wi-Fi are common among minority populations [40] and could explain why multiple participants became nonengaged and stopped submitting videos or responding to the research team shortly after they enrolled. Although providing devices and cellular data could potentially increase enrollment and engagement with electronic health interventions and ensure consistent intervention delivery, this could also add significant costs that may limit intervention sustainability and use in clinical practice. Finally, although our study suggests Mobile DOT increases hydroxyurea adherence, it is possible that the Hawthorne effect may have also contributed because participants were aware their adherence was going to be closely monitored.

### Participant Feedback

Participants’ feedback from this study provides insights into modifications that may make this and other adherence interventions more engaging, reduce attrition, and increase adherence in future studies. First, changing the frequency that medication reminders alerts are provided could reduce alert fatigue. In addition, providing feedback that notifies participants how their adherence compares with their previous adherence and with others’ could influence participants’ perception of the intervention. Incentives may be needed to reward both high adherence and incremental adherence improvements to encourage the most nonadherent participants to continue to engage with the intervention. Finally, for multidimensional interventions, quantifying each component’s impact on adherence could identify if components need to be eliminated or optimized or if additional components, such as gamification, may be needed because they might promote engagement with the entire intervention [41].

MPR was used to measure hydroxyurea adherence in this study. Although the medication event monitor system caps are considered the *gold standard* adherence measure [42], these devices were not used in this study because they are not compatible with liquid hydroxyurea formulations, which are commonly used in young children. It is important to note, however, that MPR only measures access to medication and not actual medication administration and can also be unreliable if dispensing reports are inaccurate or incomplete [21]. Engaged participants’ hydroxyurea adherence by MPR and VDOT were similar in this study, but for patients who may have accumulated a supply of hydroxyurea from past prescriptions, MPR may underestimate adherence. Ultimately, studies that validate hydroxyurea adherence measures are needed to accurately identify nonadherent children who could benefit from an adherence intervention. Furthermore, other hydroxyurea adherence interventions are under study, including an electronic alert intervention [43] and a community health care worker intervention [44], and show feasibility, and valid measures will be needed to determine which intervention is most effective.

Finally, systematic reviews suggest there are insufficient data to determine if mobile health interventions, such as Mobile DOT, are cost-effective [45,46]. Although an increasing number of insurers are already providing financial incentives to their members to encourage healthy behaviors [47], to employ insurers to provide this health behavior intervention in clinical practice, future studies will need to evaluate if it is economically feasible and sustainable. We acknowledge that the cost to improve hydroxyurea adherence may be significant, considering poor adherence is difficult to improve and sustain. However, the cost to provide this intervention could be reduced if technology improvements reduce the time and resources required for implementation and if it is reserved for patients with adherence challenges. Ultimately, we suspect that because SCD is a chronic disease that results in costly complications that are reduced with effective hydroxyurea use [48], even relatively costly adherence interventions may still be implementable, and perhaps cost-effective, in clinical practice.

### Conclusions

In conclusion, we demonstrate improved 6-month hydroxyurea adherence, MCV, and HgbF level among children with SCD who engaged with an electronic, multidimensional hydroxyurea adherence intervention. Future studies that determine how to increase patient engagement with hydroxyurea adherence interventions, how to sustain improved adherence, and how these interventions impact the outcomes of children with SCD are warranted. If successful, they may be generalizable to improve medication adherence and outcomes of other chronically ill populations.

## Abbreviations

ACS: acute chest syndrome
ANC: absolute neutrophil count
EMR: electronic medical record
HgbF: fetal hemoglobin
Hgb: total hemoglobin
HBM: Health Behavior Model
MCV: mean corpuscular volume
MPR: medication possession ratio
Mobile DOT: Mobile Directly Observed Therapy
NCH: Nationwide Children’s Hospital
SCD: sickle cell disease
SMS: short message service
VOC: vaso-occlusive crisis
